# A System of Surgery

**Published:** 1847-11

**Authors:** 


Art. VII.—*A System of Surgery.* By J. M. Chelius, Doctor in Medicine and Surgery, Public Professor of General and Ophthalmic Surgery, Director of the Chirurgical and Ophthalmic Clinic in the University of Heidelberg, etc. etc. etc. Translated from the German, and accompanied with additional Notes and Observations. By John F. South, late Professor of Surgery to the Royal College of Surgeons of England, and one of the Surgeons to St. Thomas's Hospital. Philadelphia: Lea and Blanchard. 3 vols. 8vo. 1847.

It is quite evident that German literature and science are growing in public favor, both in England and this country. The appearance of the work before us, first in London and now in Philadelphia, bears testimony to this fact. We are coming to appreciate the labors of our German brethren at their true value, and this work on surgery will do much to raise that estimate very high. The author has had ample opportunities to render it as perfect as it can be made, having elaborated it through six editions. At first, the object he had in view was the production of a work embracing a short and clear description of surgical diseases and their treatment, and referring to the best works on the subject in Germany and foreign countries, which should serve as a text-book for pupils attending his lectures. In subsequent editions deficiencies were supplied and new discoveries embraced, until the work grew to be one of the most complete in surgical literature. From 1821 to 1843, a period of little more than twenty years, six editions were called for in Germany, and in that time it was translated into six different languages. To these translations Prof. South has brought the profession of his own and this country under obligations by adding a seventh, the value of which is signally enhanced by his additions to the original.

Professor Chelius protests with characteristic good sense against the divorce of medicine and surgery. We cannot give a better idea of his sound judgment than by quoting the paragraph in which his views on this subject are expressed:

"The inadmissibility of dividing medicine from surgery is most palpable, when we endeavor to determine the object of the latter and the diseases comprehended within its boundaries, as it never can have a perfectly determined limit in opposition to the other. All diseases which are cured by the application of mechanical means have been called surgical diseases, a definition at once too narrow and too comprehensive, as many so-called medical diseases are removed only by the application of surgical means, and many diseases are evidently within the jurisdiction of surgery, which very often can be cured only by internal or external pharmaceutical means. The distinction between external and internal diseases, which has been established as the ground of division between surgery and medicine, is entirely without meaning."

Of a work so extended as this it would be useless to attempt anything like an analytical review, and in our present circumscribed limits we propose nothing more than the most general notice. From the extracts appended the reader will be able to judge of the style of the author, which he will find distinguished by clearness and brevity.

On the bites of insects and vipers he makes the following remarks:

"The severity of the symptoms after the bite of a viper depends on various circumstances—for example, whether more or less fluid has been poured from the poison bag into the wound; whether the viper was excited or not at the moment when he bit. In winter the poison is less active than in summer, but very rarely is the bite fatal. Immediately after the bite a severe burning in the wound occurs, the bitten part inflames and swells, and the inflammation spreads over the whole limb; the lymphatic vessels are red and swollen to the next glands; the glands themselves swell; high fever comes on, delirium, small pulse, vomiting, pain in the region of the heart, and often in that of the throat; not unfrequently convulsions, jaundice, anxiety of mind, fainting also are noticed.

"The best mode of destroying the poison in the wound, and for preventing its absorption, is, after cutting upon it, to cauterize it with strong liquor ammoniæ or with butter of antimony; the compression of the limb above the bitten part by means of a cord, and the applicatin of a cupping-glass. The neighborhood of the wound is to be rubbed with oil; the part may be touched with oil in which caustic ammonia has been mixed. Fluid volatile alkalies are to be given internally, and the patient kept in bed, in order to keep up properly the accompanying perspiration."

We would add, that for the sting of the bee and other insects *aqua ammoniæ* is a most effectual remedy, at once annulling the pain.

The poison of the mad-dog appears to be mucus from the lungs, and not the saliva. He says:

"The spittle (but according to Trolliet, who found the salivary glands unchanged, the mucus secreted from the inflamed mucous membrane of the bronchi) is the vehicle of the mad poison, which has been proved beyond all doubt by Hertwig's experiments. This poison is of a definite character, can impregnate various substances, and retains its activity for a long while. It need not be applied directly to an open wound to manifest its effects; it may be taken up on parts which have a very thin epidermis, even without a wound. The poison seems to remain entirely inactive when applied to the uninjured mucous membrane of the digestive organs. The contagion is also held in the blood of the mad beast, as proved by Hertwig's experiments with inoculation. Every bite of a mad beast does not produce madness; perhaps a peculiar idiosyncrasy is madness; but the bite may be also where, the part being covered with clothes, the spittle is retained in them, and the wound is not poisoned. In beasts which have become mad from contagion, the contagion is developed, and can again propagate the disease. In reference to grass-feeding beasts, the experiments have a different result. The propagation of madness from the contagion of man to beasts, and especially to dogs, is proved by experiments; but no case is known in which it has been propagated from one man to another."

Among the consequences of injuries of the head he refers to the well known affection of the liver:

"*Abscesses in the liver*, and also in other intestines of the belly, not rarely occur after previous injuries of the head. They often happen without the intestines having suffered any shock; and often they do not take place, although there has been a severe shock. They more frequently appear after injuries of the head which suppurate than after concussion without wound. They are frequently observed in affections of the brain which depend on internal causes—for instance, in chronic inflammation of the membranes, in the so-called fungous growth of the dura mater, and so on."

To which the Translator adds:

"'In the liver,' says Hennen, 'morbid appearances are found throughout every shade of affection of its membranes or its secretion, either pain and tumefaction with bilious diarrhea, or the same with a perfect torpor of its functions, and inflammatory affections, from increased vascularity to the formation of extensive collections of matter. In the spleen, pain, tumefaction, hardness and abscesses are occasionally observed. The stomach suffers more frequently than any other organ; but it appears to be more from general nervous sympathy than from any organic affection, which is seldom discovered on dissection.'

"Hennen further and very justly remarks: 'It often happens, however, that neither the liver nor any other organ seems to sympathize with the injuries of the head, while in other cases almost every viscus will appear to suffer more or less. The sympathetic affections vary in the organs which they attack and in the degree of violence. In the thorax they appear from simple increased secretion of the lungs, to tubercles and extensive purulent formation in their substance. Serum is also found in the cavity, and very frequently in the pericardium, and even in the heart itself abscesses have been discovered.'

"The same writer mentions that 'priapism is occasionally observed in wounds of the head,' and mentions a case in which, 'on dissection, the dura mater was found extensively separated all over the head. This separation included the tentorium cerebelli, and beneath its edge about four drachms of coagulated blood were found, the principal part of which lay on the cerebellum.'

"As to the 'loss of the generative faculty, and atrophy of the organs connected with it, which have been attributed to blows on the back of the head,' Hennen observes: 'The fact is certain; but whether the anti-aphrodisiac effects proceed from injury to the organs of sexual love or to a general loss of power, is a subject for future inquiry.'"

Neither of these writers refers to the theory of Budd respecting the origin of hepatic abscesses in such cases—namely, obstruction in the veins of the liver by pus globules.

This learned and valuable work is for sale at the well furnished bookstores of Messrs. Maxwell and Prescott in this city.

